# Proposed Enlargement of the Journal

**Published:** 1841-12

**Authors:** 


### PROPOSED ENLARGEMENT OF THE JOURNAL.

Such of our readers as have not yet signified their desire to receive the Journal enlarged with book matter, but may intend to do so, need not carry that intention into effect; as the limited number of returns yet made, has determined our publishers to give up the enterprise.

